# An Autism-Associated *de novo* Mutation in GluN2B Destabilizes Growing Dendrites by Promoting Retraction and Pruning

**DOI:** 10.3389/fncel.2021.692232

**Published:** 2021-07-30

**Authors:** Jacob A. Bahry, Karlie N. Fedder-Semmes, Michael P. Sceniak, Shasta L. Sabo

**Affiliations:** ^1^Department of Biology, Central Michigan University, Mount Pleasant, MI, United States; ^2^Graduate Program in Biochemistry, Cell and Molecular Biology, Central Michigan University, Mount Pleasant, MI, United States; ^3^Department of Pharmacology, Case Western Reserve University, Cleveland, OH, United States; ^4^Neuroscience Program, Central Michigan University, Mount Pleasant, MI, United States

**Keywords:** autism, neurodevelopment, GluN2B (NMDA receptor subunit NR2B), dendrite development, *GRIN2B* gene, NMDA receptor, live imaging

## Abstract

Mutations in *GRIN2B*, which encodes the GluN2B subunit of NMDA receptors, lead to autism spectrum disorders (ASD), but the pathophysiological mechanisms remain unclear. Recently, we showed that a GluN2B variant that is associated with severe ASD (GluN2B^724t^) impairs dendrite morphogenesis. To determine which aspects of dendrite growth are affected by GluN2B^724t^, we investigated the dynamics of dendrite growth and branching in rat neocortical neurons using time-lapse imaging. GluN2B^724t^ expression shifted branch motility toward retraction and away from extension. GluN2B^724t^ and wild-type neurons formed new branches at similar rates, but mutant neurons exhibited increased pruning of dendritic branches. The observed changes in dynamics resulted in nearly complete elimination of the net expansion of arbor size and complexity that is normally observed during this developmental period. These data demonstrate that ASD-associated mutant GluN2B interferes with dendrite morphogenesis by reducing rates of outgrowth while promoting retraction and subsequent pruning. Because mutant dendrites remain motile and capable of growth, it is possible that reducing pruning or promoting dendrite stabilization could overcome dendrite arbor defects associated with *GRIN2B* mutations.

## Introduction

Autism spectrum disorder (ASD) is a neurodevelopmental disorder (NDD) characterized by restricted, repetitive behavior, and social deficits. A small number of genes have been identified as having a high probability of bearing mutations that cause sporadic ASD (Abrahams et al., 2013). One of these high-confidence genes is *GRIN2B*. *GRIN2B* encodes the GluN2B subunit of NMDA receptors, ionotropic glutamate receptors that are essential for plasticity and brain development (Sanz-Clemente et al., 2013). Recently, *GRIN2B* has been identified as 1 of the 10 genes most likely to be key nodes that may control the larger network of autism risk genes (Fan et al., 2020). Homozygous *GRIN2B* knockout mice die perinatally, indicating that GluN2B is required for neural development (Kutsuwada et al., 1996).

Many *GRIN2B* mutations have been identified among individuals with ASD and other NDDs (Myers et al., 2011; O’Roak et al., 2011, 2012, 2014; Tarabeux et al., 2011; Talkowski et al., 2012; Yoo et al., 2012; De Rubeis et al., 2014; Iossifov et al., 2014; Kenny et al., 2014; Pan et al., 2015; Sanders et al., 2015; Takasaki et al., 2016; Platzer et al., 2017). However, it is not yet understood how *GRIN2B* mutations lead to pathogenesis of NDDs. Studies in non-neuronal cells have begun evaluating how *GRIN2B* mutations affect NMDAR function by examining channel properties and subcellular trafficking (Swanger et al., 2016; Liu et al., 2017; Bell et al., 2018; Fedele et al., 2018; Vyklicky et al., 2018; Li et al., 2019; Sceniak et al., 2019), but only a few *GRIN2B* mutations have been studied in neurons (Liu et al., 2017; Sceniak et al., 2019).

The first ASD-associated *GRIN2B* mutation to be discovered was a *de novo* splice site mutation (O’Roak et al., 2011) that is predicted to truncate GluN2B within the second extracellular loop (S2), which forms part of the agonist binding domain (ABD). In addition to this variant (GluN2B^724t^), four more *GRIN2B* mutations have been identified among individuals with ASD or intellectual disability that also lead to truncation within S2 (Endele et al., 2010; Kenny et al., 2014; Stessman et al., 2017). Furthermore, 32 protein-altering mutations occur in the ABD, 20 of which are within S2 (Swanger et al., 2016; Platzer et al., 2017; Stessman et al., 2017).

We recently demonstrated that GluN2B^724t^ expression causes a striking impairment in dendrite morphogenesis, leading to reduced dendrite length and complexity (Sceniak et al., 2019). This reduction in dendritic arborization was associated with a lack of mutant subunit surface trafficking and distribution into dendrites. Further, dendritic spine number was likely decreased by the presence of GluN2B^724t^ since spine density was unaltered (Sceniak et al., 2019). Dendrite morphology directly affects the extent of synaptic connectivity, the number of potential synaptic partners a neuron interacts with, and dendritic filtering of postsynaptic responses (Ledda and Paratcha, 2017; Martínez-Cerdeño, 2017). Therefore, restricting dendrite length and branching is expected to change what information is received by a neuron, as well as how that information is processed by a neuron, consistent with the hypothesis that abnormal dendrite morphogenesis leads to symptoms of ASD and ID.

Dendritic arbors are established through repeated cycles of formation of new branches followed by their elongation (Lohmann and Wong, 2005; Yoong et al., 2019). Ultimately, nascent branches are either stabilized or retracted and eliminated. Distinct molecular mechanisms underlie different aspects of dendrite growth (Vaillant et al., 2002; Baumert et al., 2020; Wilson et al., 2020). For example, glutamate-mediated signaling through mGluR5 receptors leads to Ckd5-mediated phosphorylation of delta-catenin. Interestingly, unphosphorylated delta-catenin promotes extension, whereas phosphorylated delta-catenin favors branching over extension (Baumert et al., 2020). As a result, altering glutamate signaling can shift growth from extension to branching. Therefore, it is important to define which features of dendrite growth are impaired in ASD. Here, we sought to address this issue by studying the dynamics of dendrite elongation, branching, stabilization, and pruning. Through live imaging of developing cortical neurons, we found that GluN2B^724t^ impeded dendrite development by reducing elongation and promoting dendritic pruning. These data suggest that ASD mutations contribute to ASD pathophysiology by shifting the dynamics of dendrite growth away from extension and toward branch elimination, thereby reducing dendritic arbor size and complexity and disrupting normal circuit development and function. Based on the observations presented here, it will be important to explore whether promoting dendrite outgrowth and branch stabilization can reverse the dendrite maldevelopment caused by mutant GluN2B.

## Results

**FIGURE 1:**
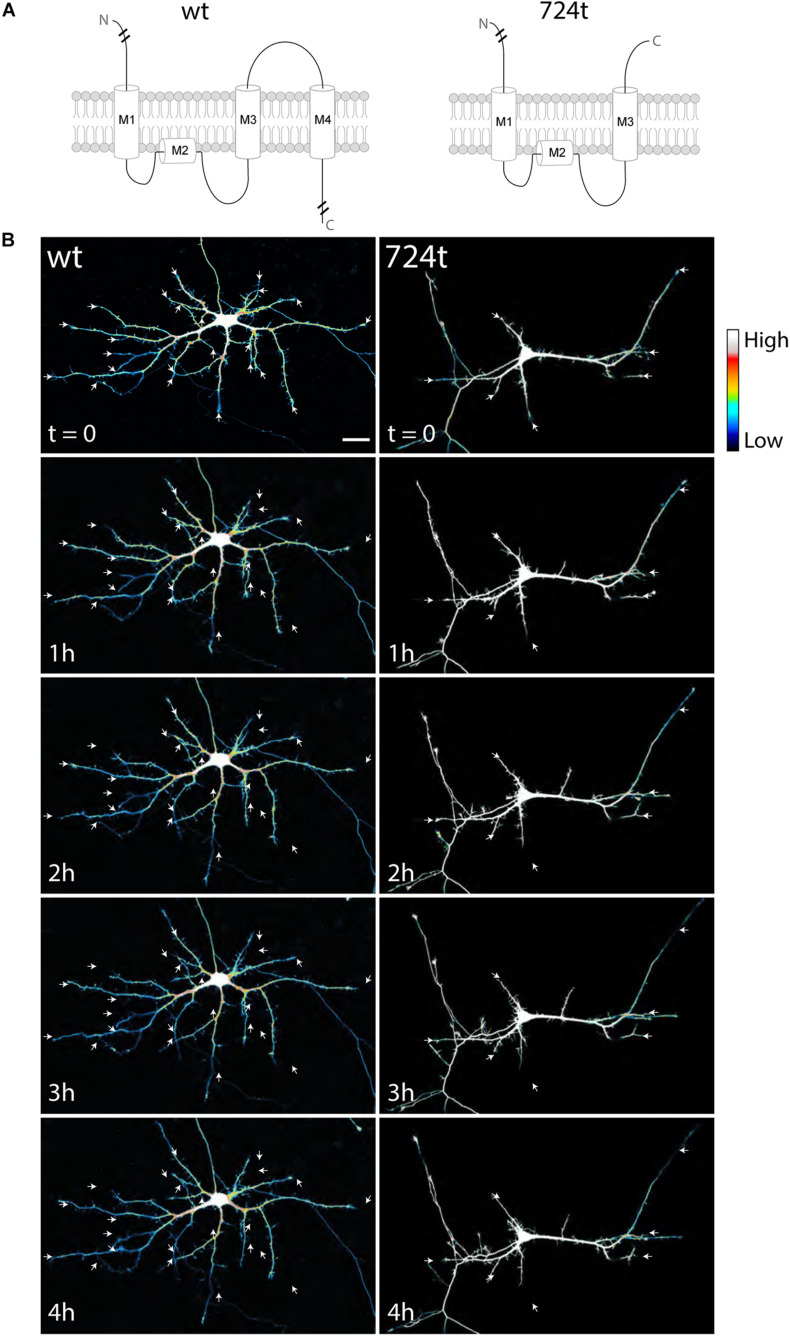
Developing neurons expressing ASD-mutant GluN2B have smaller dendrites. **(A)** Schematic of GluN2B^WT^ (*left*) and GluN2B^724t^ (*right*). M1, M2, M3, and M4 represent membrane domains. To focus on the mutation site, full amino and carboxy termini are not depicted when indicated by line breaks. **(B)** Representative images of neurons (5–9 DIV) transfected with mutant (*right)* or wildtype (*left*) GFP-tagged GluN2B (not shown) and tdTomato (color coded with an intensity scale, as indicated by the color key). Arrows point to terminal ends of dendrites. Imaging times are displayed in hours, relative to the start of imaging. Scale bar, 25 μm.

**FIGURE 2:**
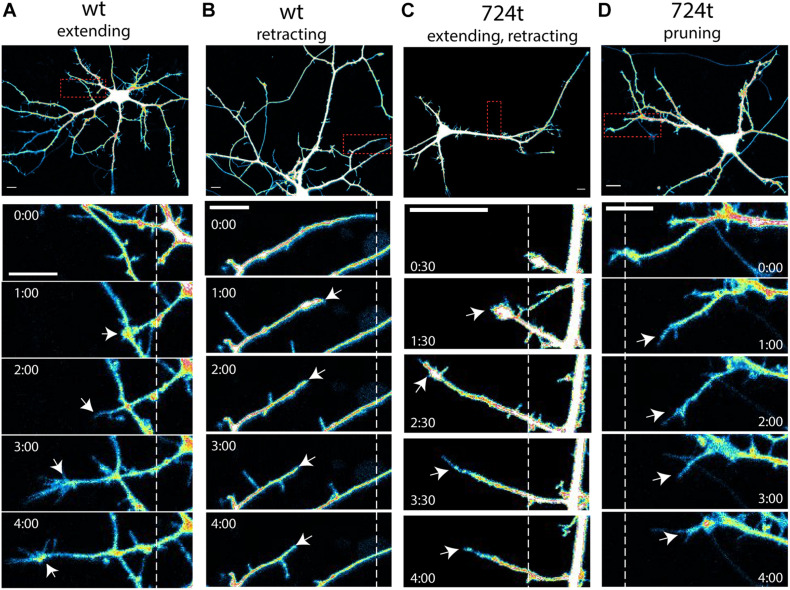
Examples of terminal dendrite dynamics. Both wild-type and mutant neurons were observed extending, retracting, branching, and pruning. **(A–D)** Representative images of dendrite behavior. *Top panels*, low magnification view of individual neurons, visualized by imaging of tdTomato cytoplasmic fills. Areas within the *red boxes* are magnified in the time-lapse panels below each neuron. *Dashed lines* indicate the positions of each dendrite tip at the beginning of imaging. *White arrows* indicate the position of the dendrite tip in each frame. Growth cones were considered the tips of dendrites, rather than individual filopodia. Imaging times are displayed relative to the start of imaging (*0:00*) and span 4 h. Intensities are colored as in Figure 1. *Scale bars*, 10 μm. **(A)** wildtype dendrite extending and remaining terminal (*white arrows*). **(B)** Wildtype dendrite retracting and remaining terminal (*white arrows*). **(C)** GluN2B^724t^ dendrite emerging, growing, and then retracting (*white arrows*). **(D)** GluN2B^724t^ dendrite retracting and pruning (*white arrows*).

To investigate how ASD-associated mutations restrict dendritic arbor size and complexity, we used live, time-lapse confocal microscopy to examine the dynamics of dendrite growth. We focused on a mutation that is predicted to truncate GluN2B in the S2 lobe of the ABD at amino acid 724 (GluN2B^724t^; O’Roak et al., 2011; Figure 1A). Rat neurons were transfected with either GluN2B^WT^ or GluN2B^724t^ tagged with GFP, along with tdTomato to fill dendrites. Imaging was performed at 5–9 days *in vitro* (DIV), a period of highly active dendrite growth and branching. Consistent with our previous observations in more mature neurons (Sceniak et al., 2019), 5–9 DIV neurons expressing GluN2B^724t^ appeared to have fewer, as well as shorter, dendrites than GluN2B^WT^ neurons (Figure 1B). Time-lapse images were collected over 4 h (Figure 1B; Supplementary Videos 1, 2), and terminal dendrites were tracked for the following fates: extension, retraction, pruning, and branching (Figure 2). Terminal dendrites were analyzed because they are the segments that are actively growing.

Here, we chose to express GluN2B^WT^ and GluN2B^724t^ on a wild-type background to reflect the heterozygous nature of patients with ASD. We previously showed that the effect of GluN2B on dendrite development was not due to overexpression of GluN2B (Sceniak et al., 2019). Furthermore, exogenous expression of *GRIN2B* does not alter the expression levels of other NMDAR subunits (Kutsuwada et al., 1996; Morikawa et al., 1998; Tang et al., 1999; Philpot et al., 2007; von Engelhardt et al., 2008), so the results reported here are likely not due to upregulation of other NMDAR subunits. It is also worth noting that the effects observed here are cell-autonomous since a small percentage (typically less than 2%) of cells express GFP-GluN2B constructs.

### ASD-Associated Mutant GluN2B Reduces Dendrite Elongation but Not Motility

Because we observed shorter dendrites in GluN2B^724t^ neurons when compared to GluN2B^WT^ neurons, we asked whether reduced dendrite length stems from either reduced elongation or increased retraction. To do so, we measured changes in dendrite length over time. For these analyses, terminal dendrites must have been greater than 7 μm over the time period analyzed. Dendrites that branched during the imaging period were not included in this analysis.

**FIGURE 3:**
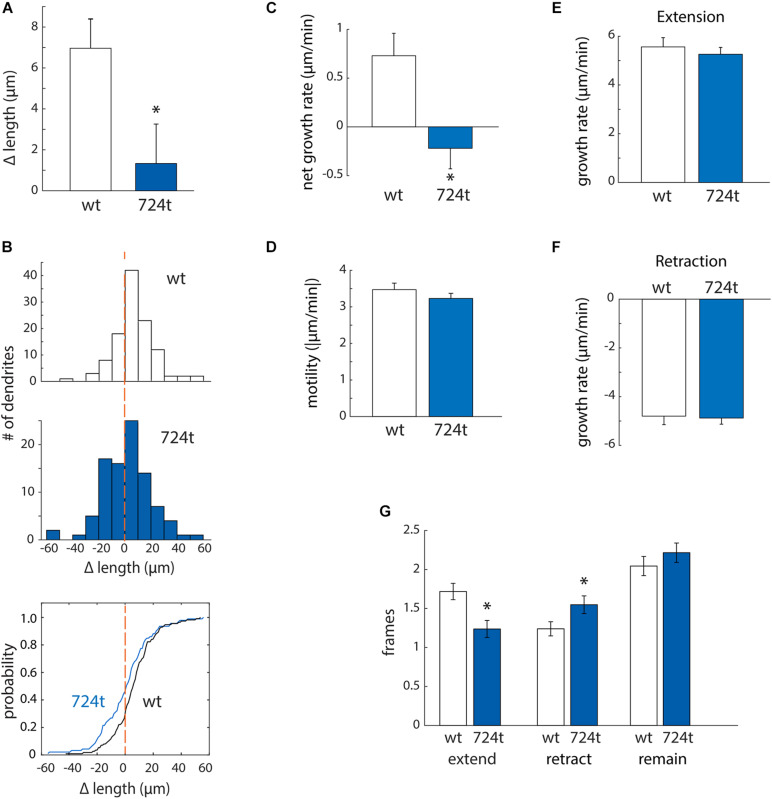
ASD-associated mutant GluN2B leads to a bias toward retraction of growing dendrites, while wild-type dendrites tend to elongate. **(A,B)** ASD-associated mutation in GluN2B disrupts dendrite growth kinetics. Quantification of the average change in length of terminal dendrites for neurons expressing GluN2B^WT^ (*white*) or GluN2B^724t^ (*blue*) from eight independent experiments. **(A)** Over 4 h, neurons expressing GluN2B^724t^ had reduced net growth. Data represent means ± S.E. (*p = 0.018, *n* = 14 GluN2B^WT^ neurons, and 14 GluN2B^724t^ neurons). **(B)** ASD-associated mutation in GluN2B biases net growth of dendrites toward retraction and away from extension. *Top and middle panels*, histograms represent the number of dendrites that lengthen or shorten, separated into bins of 10 μm, with negative values representing a terminal dendrite becoming shorter over 4 h of live imaging. *Bottom panel*, cumulative probability distribution of changes in dendrite length. GluN2B^WT^ (*n* = 113 dendrites), and GluN2B^724t^ (*n* = 93 dendrites). *Dashed orange lines*, zero change in length. **(C,D)** ASD-mutant GluN2B reduces the net rate of dendrite outgrowth but not overall motility of terminal dendrites. Lengths of terminal dendrites were measured every 12 min for 1 h. Data represent means ± S.E. (*n* = 113 GluN2B^WT^ dendrites, and 93 GluN2B^724t^ dendrites). **(C)** ASD-associated mutant dendrites elongated at a reduced rate, compared to neurons expressing GluN2B^WT^ (*p = 0.0008). **(D)** Overall motility of terminal dendrites was similar for neurons transfected with either wildtype or mutant GluN2B (*p* = 0.7147). Motility represents all movements, regardless of direction, and was quantified by averaging the absolute values of instantaneous growth rates. Rates of extension [**(E)**, *p* = 0.5255] and retraction [**(F)**, *p* = 0.3121] were similar in ASD and WT neurons. Time spent extending [**(G)**, left, *p* = 0.0017] and retracting [**(G)**, middle, *p* = 0.0283] were significantly different, however. Time spent not actively extending or retracting was similar for ASD and WT neurons [**(G)**, right, *p* = 0.2138]. Each frame represents 12 min.

First, to determine whether mutant dendrites grow at a different net rate than wildtype dendrites, we traced terminal dendrites in the first and last frames of the movies. GluN2B^724t^ dendrites averaged a significantly smaller net gain in length than GluN2B^WT^ dendrites (Figure 3A; GluN2B^WT^: 6.87 ± 1.48 μm, *n* = 113 dendrites from 14 cells and GluN2B^724t^: 1.14 ± 1.75 μm, *n* = 93 dendrites from 14 cells; *p* = 0.018). In addition, more mutant dendrites netted a loss of length (Figure 3B). To further delineate the mechanisms of reduced net growth of mutant neurons, we analyzed the instantaneous growth rate of terminal dendrites. By examining the instantaneous growth rate, we could remove contaminating effects of periods when dendrites were neither extending nor retracting since only frames with detectable movement were analyzed. Consistent with what we observed over 4 h, GluN2B^724t^ dendrites elongated more slowly (Figure 3C; GluN2B^WT^: 0.73 ± 0.23 μm/min, *n* = 565 movements from 113 dendrites and GluN2B^724t^: -0.22 ± 0.21 μm/min, *n* = 465 movements from 93 dendrites; *p* = 0.0008). However, the absolute values of the instantaneous rate of change for wildtype and mutant neurons were similar (Figure 3D; GluN2B^WT^: 3.47 ± 0.18 μm/min, *n* = 565 movements from 113 dendrites and GluN2B^724t^: 3.23 ± 0.14 μm/min, *n* = 465 movements from 93 dendrites; *p* = 0.7147), indicating that GluN2B^724t^ dendrites were not less motile than GluN2B^WT^ dendrites.

Mutant dendrites could have reduced growth either: (i) because elongation is decreased or (ii) because retraction is increased. To distinguish between these possibilities, we separately analyzed extension and retraction. First, we evaluated rates of extension and retraction. The average rate of extension did not differ for wildtype and mutant dendrites (Figure 3E; GluN2B^WT^: 5.56 ± 0.38 μm/min, *n* = 194 movements from 113 dendrites and GluN2B^724t^: 5.26 ± 0.28 μm/min, *n* = 115 movements from 93 dendrites; *p* = 0.5255). The rates of retraction for both GluN2B^WT^ and GluN2B^724t^ were also similar (Figure 3F; GluN2B^WT^: -4.80 ± 0.35 μm/min, *n* = 140 movements from 113 dendrites and GluN2B^724t^: -4.88 ± 0.25 μm/min, *n* = 144 movements from 93 dendrites; *p* = 0.3121). Given that net growth was substantially reduced in mutant neurons (Figures 3A,C) while rates of extension and retraction were similar (Figures 3E,F), these data suggest that growing dendrites must spend less time extending and/or more time retracting in neurons expressing GluN2B^724t^. We found that both of these changes occur (Figure 3G; GluN2B^WT^: 1.72 ± 0.11 frames extending, 1.24 ± 0.09 frames retracting, and 2.04 ± 0.12 frames not changing length; GluN2B^724t^: 1.24 ± 0.11 frames extending, 1.548 ± 0.11 frames retracting, 2.22 ± 0.12 frames not changing; *p* = 0.0017, 0.0283, and 0.2138, respectively). Therefore, expression of GluN2B^724t^ shifts dendrite dynamics away from extension and toward retraction, leading to shorter dendrite arbors.

### GluN2B^724t^ Expression Promotes Pruning of Dendrite Branches

**FIGURE 4:**
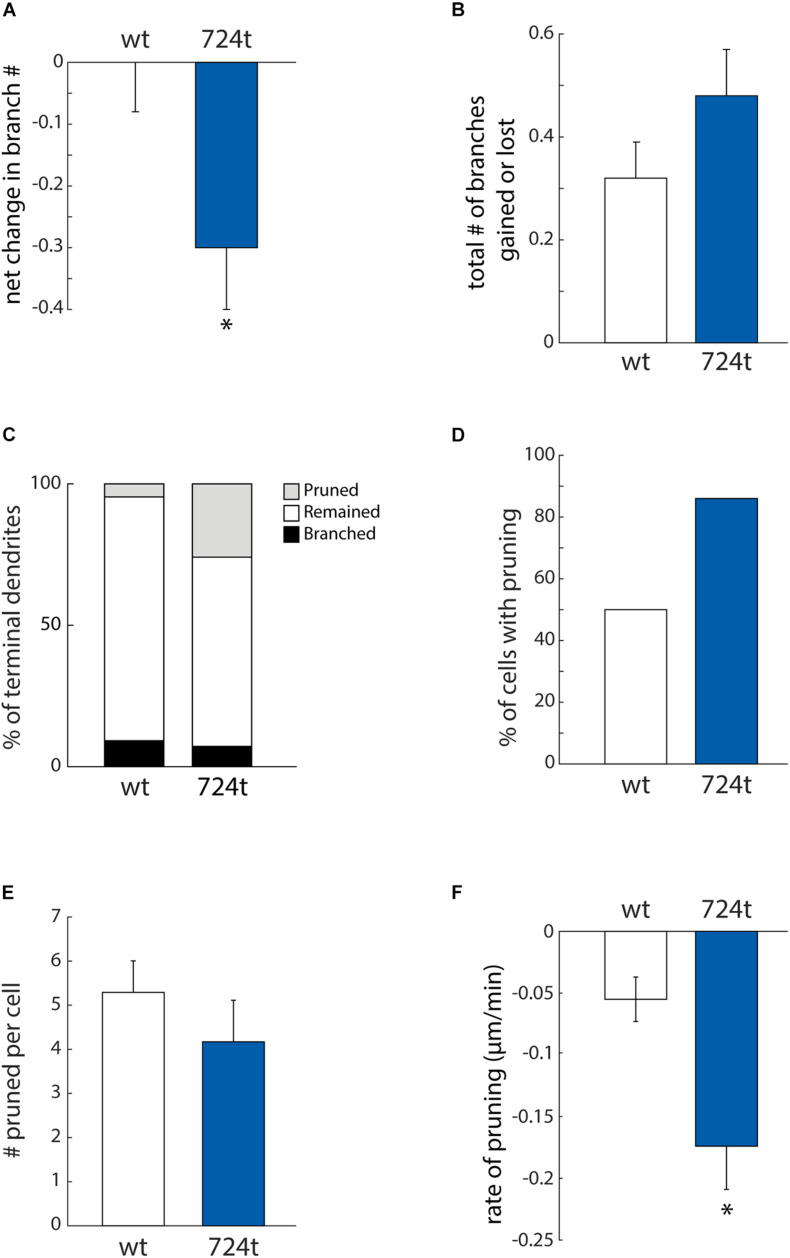
ASD-associated mutation in GluN2B increases dendrite pruning but does not affect formation of new branches. **(A)** ASD mutant neurons (*blue bars*) display a net loss of terminal dendrite branches, while wild-type neurons (*white bars*) have a stable number of terminal dendrites. Positive values represent gain of dendrite branches, while negative values represent loss of dendrite branches. Data represent the mean ± S.E. (*p = 0.0302, *n* = 14 GluN2B^WT^, and 14 GluN2B^724t^ neurons). **(B)** To calculate the total change in the number of terminal dendrites per cell per hour, both gains and losses of dendrites are represented as positive values. The ASD-associated mutation did not have a significant impact on the total change in the number of terminal dendrites (*p* = 0.2553). **(C)** Outcomes for terminal dendrites. A higher percentage of ASD-mutant dendrites were pruned, while a higher percentage of wild-type dendrites remained stable as terminal dendrites. The percentages of dendrites that branched were similar for mutant and wild-type dendrites. **(A–C)** For each cell, data were collected every hour for 4 h. **(D)** An increased percentage of ASD-associated mutant neurons had at least one pruned terminal dendrite over a 4-h time period, as compared to wild-type neurons. **(E)** Neurons that had at least one pruned dendrite had a similar number of pruned dendrites. Data represent the mean ± S.E. (*p* = 0.114, *n* = 7 GluN2B^WT^, and 12 Glun2B^724t^ neurons). **(F)** Pruned dendrites retracted at a significantly faster rate for neurons expressing GluN2B^724t^ compared to neurons expressing GluN2B^WT^ (*p* = 0.008, *n* = 37 GluN2B^WT^, and 50 GluN2B^724t^ dendrites).

Neurons expressing GluN2B^724t^ also have fewer branches (Sceniak et al., 2019). To determine whether we could capture that change in branching in our imaging time period, we analyzed the net change in the number of terminal dendrites. Within this analysis, positive values corresponded to dendrites gained from branching, and negative values represented pruned dendrites. Mutant neurons tended to lose dendrite branches, whereas wild-type neurons did not (Figure 4A; GluN2B^WT^: 0.00 ± 0.08, *n* = 14 cells and GluN2B^724t^: -0.30 ± 0.10, *n* = 14 cells; *p* = 0.0302). Although there was a net loss of branches in the mutant, these dendrites were still dynamic since the mean of the absolute values of all branch gains and losses was not significantly different in mutant and wild type neurons (Figure 4B; GluN2B^WT^: 0.32 ± 0.07, *n* = 14 cells and GluN2B^724t^: 0.48 ± 0.09, *n* = 14 cells; *p* = 0.2553).

Apical and basal dendrites of pyramidal cells are differentially affected by mutation or loss of two autism-associated genes, Epac2 and TAO2 (de Anda et al., 2012; Srivastava et al., 2012). This raises the question of whether apical and basal dendrites are similarly affected by GluN2B^724t^. To test this, we quantified the net change in number of terminal dendrite branches for only the principal/apical dendrites. In wild-type and mutant neurons, a similar percentage of the total population of terminal dendrites belonged to the principal dendrite arbor (GluN2B^WT^: 67.74 ± 0.09%, *n* = 13 cells and GluN2B^724t^: 60.69 ± 0.08%, *n* = 13 cells; *p* = 0.5378). Interestingly, the stability of apical branches did not differ when comparing wild-type and mutant neurons: in 4 h of imaging, the same proportion of wild-type and mutant GluN2B neurons lost apical branches (2 out of 13 neurons for both GluN2B^WT^ and GluN2B^724t^) GluN2B^WT^. In addition, apical dendrites gained 1.15 ± 0.42 branches (*n* = 13 cells), and GluN2B^724t^ apical dendrites gained 0.46 ± 0.39 branches (*n* = 13 cells; *p* = 0.2787), even though the mutant neurons tended to lose dendrite branches in the same movies (Figure 4A).

The observed reduction in dendrite branching could stem from either reduced formation of new branches or increased elimination of existing branches. To distinguish between these two possibilities, we analyzed branching and pruning of individual dendrites. Dendrites that existed at the start of recording could have one of three possible fates: branch, prune, or remain terminal (i.e., neither branch nor prune). Within our 4 h imaging period, a higher percentage of GluN2B^724t^ dendrites pruned (Figure 4C; wt: 4.6%, *n* = 131 and 724t: 25.9%, *n* = 139 dendrites). Conversely, a lower percentage of mutant dendrites remained terminal (Figure 4C; GluN2B^WT^: 86.3%, *n* = 131 and GluN2B^724t^: 66.9%, *n* = 139). Interestingly, rates of formation of new branches were similar in wild-type and mutant neurons (Figure 4C; GluN2B^WT^: 9.2%, *n* = 131 and GluN2B^724t^: 7.2%, *n* = 139). These data suggest that dendrites of neurons transfected with GluN2B^724t^ were less stable and had an increased bias toward pruning.

The observed bias toward pruning could be from more cells having dendrites that prune, or from more dendrites pruning per cell. We found that a higher percentage of mutant neurons had dendrites that pruned (Figure 4D; GluN2B^WT^: 50%, *n* = 14 cells and GluN2B^724t^: 86%, *n* = 14 cells). Of the neurons that had pruned dendrites, there was no difference in the number of transient dendrites per cell [Figure 4E, 5.29 ± 0.71 (wt) and 4.17 ± 0.94 (724t); *n* = 7 GluN2B^WT^ and 12 GluN2B^724t^ neurons; *p* = 0.114]. This shows two things: (1) more neurons expressing GluN2B^724t^ had pruned dendrites, and (2) of the cells that had pruned dendrites, neurons expressing mutant and wildtype GluN2B had a similar number of dendrites that pruned.

For the dendrites that pruned, we next asked whether the pruning process occurred more rapidly in GluN2B^724t^ expressing neurons by analyzing the rate of elimination of pruned branches. All imaging frames were included in the calculations, including frames with advances or no movement. The rate of elimination was increased for GluN2B^724t^-expressing neurons compared to GluN2B^WT^-expressing neurons [Figure 4F; -0.055 ± 0.018 μm/min (GluN2B^WT^), -0.174 ± 0.035 μm/min (GluN2B^724t^); *p* = 0.008; *n* = 37 GluN2B^WT^ dendrites from seven neurons and 50 GluN2B^724t^ dendrites from 12 neurons]. Together, our data indicate that ASD mutant GluN2B destabilizes growing dendrites, leading to increased rates of branch pruning, which ultimately leads to reduced arbor complexity.

## Discussion

Despite abundant evidence that GluN2B influences dendrite growth and arborization (Ewald et al., 2008; Espinosa et al., 2009; Sepulveda et al., 2010; Bustos et al., 2014; Keith et al., 2019), the specific role of GluN2B in the dynamics of dendrite outgrowth and branching remains poorly understood. In the Xenopus tectal system, GluN2B over-expression did not alter branch additions or retractions but increased the appearance of transient dendrites, defined as existing no longer than 2 h (Ewald et al., 2008). Here, we found that rodent cortical neurons expressing GluN2B^724t^ had more retraction events, fewer stable dendrites, and more transient branches than wild-type neurons. In our experiments, transient dendrites corresponded to dendrites that existed at the start of imaging but did not persist for the entire 4 h imaging period. Although transient dendrites are defined somewhat differently in these two studies, both point to a role for GluN2B in regulation of dendrite turn-over and stability. Overall, these data suggest that ASD-associated *GRIN2B* mutations may disrupt normal GluN2B-dependent stabilization of nascent dendrites. GluN2B^724t^ may restrict dendrite outgrowth and branching by either inhibiting downstream signals that stabilize growing dendrites or by activating signaling pathways that promote retraction and pruning.

In cortical pyramidal neurons, apical and basal dendrites are distinct subcellular compartments. Here, we found that ASD mutant GluN2B lead to a net loss of terminal dendrites. However, in the same movies, apical/principal dendrite branches were unaffected by GluN2B^724t^, indicating that apical and basal dendritic arbors are differentially affected by GluN2B^724t^. Our observations are consistent with previous studies demonstrating that basal dendrite arbors are specifically reduced by expression of ASD-associated mutations in Epac2 (Srivastava et al., 2012) and loss of the ASD-associated TAO2 (de Anda et al., 2012). Interestingly, it was recently shown that GluN2B antagonists preferentially reduced basal dendrite branch number without significantly changing apical dendrite branching in cortical pyramidal neurons in organotypic slices (Gonda et al., 2020).

The synaptotrophic hypothesis posits that dendritic arbors are stabilized and shaped by concomitant synapse formation (Vaughn, 1989; Wong and Ghosh, 2002; Cline and Haas, 2008; Valnegri et al., 2015). This hypothesis is supported by observations that, in *Xenopus* tectal neurons, strong synapses are associated with stabilized dendrites, while weak synapses are not (Wu et al., 1999). Supporting a role for NMDARs in mediating synaptotrophic dendrite growth in mammalian cortex, NMDAR activation stabilizes stellate cell dendrites that form synapses with appropriate presynaptic partners (Mizuno et al., 2014). In this study, local activation of synaptic NMDARs was proposed to stabilize dendrite branches and promote their elongation, while global NMDAR signaling reduced dendrite extension and decreased the motility of terminal dendrite branches throughout the dendritic arbor.

It seems likely that the impaired dendrite elongation and branching that we observed in ASD mutant neurons is coupled to reduced synaptogenesis. Here, we observed that terminal dendrites are less stable and more likely to be eliminated in neurons transfected with GluN2B^724t^ than with GluN2B^WT^. We previously showed that dendrites of mutant neurons form fewer spine synapses (Sceniak et al., 2019), but we do not yet know whether the number of non-spine synapses formed with dendrite shafts is similarly reduced. On one hand, if synaptotrophic mechanisms couple dendrite growth and synaptogenesis, then reduced stability of terminal dendrites and increased pruning could be downstream of reduced synaptogenesis. In addition, mutant NMDARs could reduce synapse strength, contributing to a deficit in dendrite stabilization. On the other hand, abnormal dendrite growth dynamics might drive the loss in synapse number in GluN2B^724t^ neurons: smaller, simplified dendrites may encounter fewer appropriate synaptic partners as they grow, driving a reduction in synapse formation.

The etiology of autism remains unclear; although, it is believed that autism is related to defects in neuronal connectivity in higher-order association areas (Geschwind and Levitt, 2007). Magnetic resonance imaging has provided evidence of both large-scale and small-scale changes in connectivity (Maximo et al., 2014), supporting this theory. Most research examining the cellular basis of connectivity abnormalities in ASD has focused on spine number and morphology or synaptic function (Kelleher and Bear, 2008; Bourgeron, 2009; Phillips and Pozzo-Miller, 2015; Varghese et al., 2017). However, it remains unclear how mutations in ASD-associated proteins cause changes in connectivity. One hypothesis is that morphological abnormalities in dendritic arbors lead to altered connectivity (Kulkarni and Firestein, 2012; Copf, 2016). Consistent with this hypothesis, our results suggest that in forms of ASD characterized by lower synapse number, ASD mutations may contribute to reduced connectivity by impairing dendrite outgrowth and stabilization.

Understanding how ASD mutations alter dendrite growth and stabilization is essential: if the bias in dendrite growth dynamics can be shifted toward outgrowth and stabilization, perhaps dendrite architecture can be restored. Because diagnosis of ASD often occurs after dendrite development is complete, it will be interesting to determine whether dendrite defects can be reversed later in development, after diagnosis of ASD.

## Materials and Methods

All studies were conducted with an approved protocol from the Case Western Reserve University Institutional Animal Care and Use Committee or the Central Michigan University Institutional Animal Care and Use Committee, in compliance with the National Institutes of Health guidelines for care and use of experimental animals.

### Neuronal Cell Culture and Transfection

Neurons and astrocytes were derived from cortices of wild-type Sprague Dawley rats at 0–1 days postnatal. Neurons were grown on a confluent monolayer of astrocytes at 5% CO_2_. Astrocytes were plated on 18 mm coverslips made of German glass (Carolina Scientific) that were acid cleaned then coated with a mixture of collagen and poly-D-lysine and grown in MEM without phenol red (Gibco), supplemented with N2 (Gibco), 10% fetal calf serum (Gibco or HyClone), 20% glucose, glutamax (Gibco), and Primocin (Invivogen). When astrocytes formed a confluent monolayer, neurons were plated on top of the astrocytes. Co-cultures were maintained in neuronal medium: Neurobasal-A (Gibco) with B27 (Gibco) and without antibiotics or phenol red, as previously described (Bury and Sabo, 2011, 2014; Sceniak et al., 2012). Half of the medium was replaced with fresh, equilibrated neuronal medium every 3–5 days.

Neurons were co-transfected with pEGFP-GluN2B constructs (2.6 μg DNA/coverslip) along with tdTomato (0.4 μg DNA/coverslip) at 2 DIV using the calcium phosphate method (Berry et al., 2012; Sceniak et al., 2019). pEGFP-GluN2B constructs were previously described (Sceniak et al., 2019). EGFP is inserted in the amino-terminal extracellular domain after the signal peptide. tdTomato-N1 was Addgene plasmid 54642.

### Live Imaging of Neurons

Neurons were imaged live at 5–9 DIV. A coverslip was transferred to a sterile, closed imaging chamber (Warner Instruments) and bathed in warmed (37°C) Hibernate-A low-fluorescence medium (Gibco), supplemented with glutamax (Gibco). The chamber was mounted on the microscope stage and enclosed in a custom-made warming box (32–35°C). GFP was used to identify cells that expressed GluN2B constructs. Large neurons with pyramidal somas, based on tdTomato fills, were chosen for imaging. Only healthy cells were imaged, as determined by the appearance of the soma under DIC and a smooth axon without fragmentation or blebbing. Transfection efficiency was sufficiently low that individual filled neurons could be imaged without interference from nearby neurons. Imaging was with a Nikon C1 Plus confocal system, Eclipse Ti-E microscope, 20X (NA 0.75) Plan Apo objective, and 590/50 nm bandpass filter to visualize tdTomato fills. A perfect focus system was used to maintain focus. Eighty images were collected at 3-min intervals over 4 h. Scanning frame times were kept minimal at 14.7 s per image. Gains were set to clearly visualize thin dendrites while minimizing saturation in thicker dendrites and were kept constant during imaging. Images were taken with pixel sizes ranging from 205.1 to 250.3 nm.

### Dendrite Analysis

Fiji (ImageJ 2.0.0-rc-65/1.51w) was used to track dendrite growth and branching, based on the tdTomato fills. Individual terminal dendrite branches were traced throughout the movies for further quantification, as described in detail below for each measure presented. Analysis was limited to neurons that remained healthy throughout the 4 h imaging period. Dendrites were identified based on their morphology (shorter than the axon, tapered). MATLAB was used to plot the data. Since the data were not normally distributed (not shown), statistical comparisons were made using Wilcoxon rank sum tests in MATLAB.

To quantify the change in length of terminal dendrites, dendrites longer than 7 μm were traced at the start (*t* = 0 h) and end (*t* = 4 h) of imaging. To calculate a net change in length, the difference between starting and ending lengths was determined, then these values were averaged for each neuron. Statistical analysis was performed using the average of each neuron. Histograms represent data for individual dendrites.

To determine instantaneous growth rates, terminal dendrites greater than 7 μm long were traced every 12 min for 1 h. This analysis interval was sufficient to capture all quantifiable movements. The instantaneous net growth rate corresponds to the average rate of change in length for all movements. The instantaneous motility corresponds to the mean of the absolute values of instantaneous growth rates. The rate of retraction was calculated by averaging growth rates for all movements resulting in loss of at least 2 μm of dendrite in 12 min. Similarly, the rate of extension is the mean of movements that added at least 2 μm of dendrite length in 12 min.

Pruning dendrites were defined as dendrites that existed at the start of imaging but retracted until no longer visible. Branching dendrites were defined as dendrites that existed at the start of imaging but became branched into 2 or more dendrites during imaging. Dendrites that remained terminal were dendrites that may have gained or lost length but never disappeared or branched. For analysis of the net change in the number of terminal dendrites, the total number of terminal dendrites per neuron was recorded each hour for 4 h. For apical dendrite analysis, the first and last frames of movies were compared for addition or loss of terminal dendrites.

To determine retraction rates for pruned dendrites, we recorded the length of each dendrite every 12 min until the dendrite was completely gone. Changes in length were divided by 12, and every pruned branch was treated as independent for statistical analysis. Transient dendrites were not considered fully pruned until they completely disappeared. This ensured that we did not include dendrites that might eventually regrow in the analysis of pruning.

## Data Availability Statement

The raw data supporting the conclusions of this article will be made available by the authors, without undue reservation.

## Ethics Statement

The animal study was reviewed and approved by Case Western Reserve University Institutional Animal Care and Use Committee and Central Michigan University Institutional Animal Care and Use Committee.

## Author Contributions

KF-S collected data and edited the manuscript. MS analyzed data and edited the manuscript. JB and SS analyzed data and wrote the manuscript. All authors contributed to the article and approved the submitted version.

## Conflict of Interest

The authors declare that the research was conducted in the absence of any commercial or financial relationships that could be construed as a potential conflict of interest.

## Publisher’s Note

All claims expressed in this article are solely those of the authors and do not necessarily represent those of their affiliated organizations, or those of the publisher, the editors and the reviewers. Any product that may be evaluated in this article, or claim that may be made by its manufacturer, is not guaranteed or endorsed by the publisher.

## Supplementary Material

Supplementary Video 1 Representative movie example of a GluN2B^WT^ GFP-GluN2B neuron. Neuron was six DIV. Movie spans 4-h of imaging. Images were collected every 3 min. Frame time was 14.718 s. This neuron is also shown in Figure 1B. Scale bar, 25 μm.

Supplementary Video 2 Representative movie example of a GluN2B^724t^ GFP-GluN2B neuron. Neuron was seven DIV. Movie spans 4-h of imaging. Images were collected every 3 min. Frame time was 14.718 s. This neuron is also shown in Figure 1B. Scale bar, 25 μm.
