# Corrigendum: An Autism-Associated *de novo* Mutation in GluN2B Destabilizes Growing Dendrites by Promoting Retraction and Pruning

**DOI:** 10.3389/fncel.2022.892217

**Published:** 2022-04-25

**Authors:** Jacob A. Bahry, Karlie N. Fedder-Semmes, Michael P. Sceniak, Shasta L. Sabo

**Affiliations:** ^1^Department of Biology, Central Michigan University, Mount Pleasant, MI, United States; ^2^Graduate Program in Biochemistry, Cell and Molecular Biology, Central Michigan University, Mount Pleasant, MI, United States; ^3^Department of Pharmacology, Case Western Reserve University, Cleveland, OH, United States; ^4^Neuroscience Program, Central Michigan University, Mount Pleasant, MI, United States

**Keywords:** autism, neurodevelopment, GluN2B (NMDA receptor subunit NR2B), dendrite development, *GRIN2B* gene, NMDA receptor, live imaging

The original article incorrectly did not include an Acknowledgments statement. The Acknowledgments Statement appears below.

We thank members of the Sabo lab, Mark Wacker and Mallary Greenlee-Wacker for helpful discussions. We thank Kellie Gallo for assistance with image analysis and for comments on the manuscript.

The authors apologize for this error and state that this does not change the scientific conclusions of the article in any way. The original article has been updated.

## Publisher's Note

All claims expressed in this article are solely those of the authors and do not necessarily represent those of their affiliated organizations, or those of the publisher, the editors and the reviewers. Any product that may be evaluated in this article, or claim that may be made by its manufacturer, is not guaranteed or endorsed by the publisher.

